# Determination of Urolithin A in Health Products by Ultra-High-Performance Liquid Chromatography

**DOI:** 10.3390/molecules30051141

**Published:** 2025-03-03

**Authors:** Yue E, Zhuang Wang, Jiahui Nie

**Affiliations:** 1 School of Chemical Engineering and Technology, Tianjin University, Tianjin 300072, China; z_wang@tju.edu.cn; 2 Zhejiang Institute of Tianjin University, Shaoxing 312300, China

**Keywords:** urolithin A, health products, ultra-high-performance liquid chromatography method

## Abstract

This study establishes and validates a novel ultra-high-performance liquid chromatography (UHPLC) method for the determination of urolithin A content in health products, a bioactive compound with potential anti-aging properties. Given the lack of standardized analytical methods for urolithin A in health products, this research addresses a critical gap in quality control. The method employs a methanol–water mobile phase, optimized gradient elution, and a specialized UPLC column (ACQUITY UPLC CSH Fluoro Phenyl) to achieve high resolution and specificity in the separation of urolithin A from its impurities. A variety of diluents, extraction solvents, and extraction times were tested to maximize analyte recovery and stability, with pure methanol yielding the highest recovery rate (over 95%) in 30 min. The method was validated in terms of linearity, sensitivity, repeatability, specificity, and precision. The calibration curve for urolithin A exhibited excellent linearity (r^2^ = 0.9998) over a concentration range of 0.100–10.000 µg/mL. Detection and quantification limits were found to be 0.051 µg/mL and 0.103 µg/mL, respectively. Precision testing revealed an inter-operator RSD of 1.3%, and recovery rates for spiked samples consistently fell within the 98–102% range. The developed method was successfully applied to analyze the urolithin A content in a commercially available health product, demonstrating its practicality for routine quality control. However, this method may currently be affected by the excipient matrix. This research contributes to the establishment of robust, reliable, and high-sensitivity analytical methods for the bioactive compounds found in health products, with significant implications for regulatory compliance and consumer safety.

## 1. Introduction

Urolithin A, a polyphenolic compound derived from the metabolism of ellagitannins by gut microbiota, has garnered significant attention due to its promising health benefits [1,2]. Primarily obtained through the microbial transformation of ellagic acid from fruits such as pomegranates, raspberries, and strawberries, urolithin A represents an important bioactive compound in human nutrition [3,4,5,6]. As research continues to highlight its potential, the compound’s metabolic pathways, biological activities, and health-promoting properties are becoming increasingly evident. The diverse pharmacological effects of urolithin A, including its anti-aging, anti-inflammatory, and anti-cancer properties, have spurred interest in its inclusion as a functional ingredient in health supplements and nutraceuticals [2,3]. However, despite these promising attributes, the lack of standardized and reliable methods for its quantification in health products remains a significant hurdle for regulatory bodies and the supplement industry [7].

The metabolism of ellagitannins into urolithin A occurs in the human gut, where specific strains of gut microbiota—such as Gordonibacter species and certain Lactobacillus strains—are responsible for the conversion of ellagic acid into urolithins [1]. This biotransformation not only affects the bioavailability of urolithin A but also its potential efficacy in promoting human health. Research has shown that urolithin A exhibits several pharmacological properties, including mitochondrial biogenesis, anti-inflammatory effects, and protection against oxidative stress [8,9]. These activities are largely attributed to its ability to enhance mitophagy, a form of selective autophagy process that clears damaged mitochondria, thus improving mitochondrial function and longevity. Studies have shown that urolithin A can promote mitochondrial autophagy, prolong the lifespan of experimental animals, or enhance muscle function [10]. Additionally, preclinical studies indicate that urolithin A may play a protective role against neurodegenerative diseases, including Alzheimer’s disease, by improving cognitive function and reducing amyloid plaque accumulation in the brain [11]. Furthermore, the compound also shows promise in cancer research, with studies highlighting its ability to inhibit cell proliferation in various cancer types, including prostate, breast, and colorectal cancers. These findings emphasize the broader therapeutic potential of urolithin A as a natural agent with anti-aging and anti-cancer properties [5,12].

Given its beneficial effects, urolithin A has found a place in the dietary supplement market, where it is marketed as an anti-aging and immunity-boosting agent [13]. The increasing demand for urolithin A supplements, particularly in regions such as North America and Europe, is a clear indicator of its potential market value. In the United States, urolithin A has been approved by the FDA for use in dietary supplements, marking an important milestone for its commercial application. Health products containing urolithin A are commonly found in capsule, powder, and liquid forms, and they are marketed as functional foods designed to combat aging, improve metabolic function, and support overall well-being [14]. Some of these products are incorporated into food matrices such as yogurt, oatmeal, and meal replacement shakes. The bioavailability of urolithin A in these products is a topic of ongoing research, as the compound’s absorption and metabolism are dependent on individual gut microbiota composition, leading to inter-individual variability in response to supplementation [1,3,15].

However, the rapid growth of the urolithin A market has highlighted a critical gap in the regulatory oversight of its content in health products, particularly in countries like China, where urolithin A is increasingly used in functional foods. At present, there are no national standards or industry-specific methods in China for the determination of urolithin A content in these health products, which raises concerns regarding product quality, consistency, and safety. The absence of standardized analytical methods poses a significant challenge for the quality control and regulatory approval of such supplements. Traditional methods, such as high-performance liquid chromatography (HPLC), are time-consuming and often fail to achieve optimal separation, leading to inaccurate results [1,16]. As a result, there is a pressing need for more efficient, reliable, and high-throughput analytical techniques that can be used for the accurate determination of urolithin A in commercial health products.

To address these challenges, this study proposes the development of a method using ultra-high-performance liquid chromatography (UHPLC) for the quantification of urolithin A in health products. The choice of UHPLC is based on its superior separation efficiency, faster analysis time, and higher resolution compared to conventional HPLC systems [17,18]. This validation protocol is designed based on the International Council for Harmonisation of Technical Requirements for Pharmaceuticals for Human Use (ICH)’s Q2 (R1) *Validation of Analytical Methods* to systematically validate parameters such as specificity, linearity, precision, accuracy, limit of detection (LOD), and limit of quantification (LOQ) to ensure the accuracy, reliability, and reproducibility of the analysis results. The validation of this method will provide reliable data on the concentration of urolithin A in health products, contributing to the establishment of national standards for the analysis of this compound in health supplements.

This study is intended to provide a robust, sensitive, and reproducible analytical method for the quantification of urolithin A in various health product formulations. The proposed method will be applied to the analysis of commercially available supplements, allowing for the determination of urolithin A content and the assessment of its consistency across different products. By providing a reliable analytical technique, this work aims to support the development of standardized testing protocols for urolithin A, which will enhance the safety and quality of health products in the market. Furthermore, this method provides a reliable tool for the quality control of urolithin A, which can support its bioavailability and pharmacokinetic studies in the future.

## 2. Results and Discussion

### 2.1. Chromatographic Conditions

#### 2.1.1. Gradient Elution Optimization

**Figure 1:**
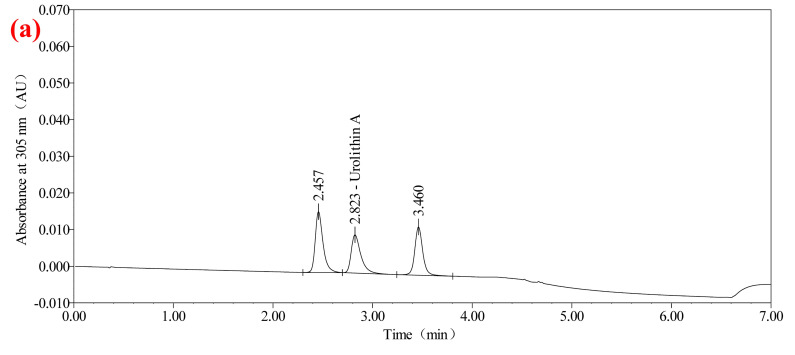

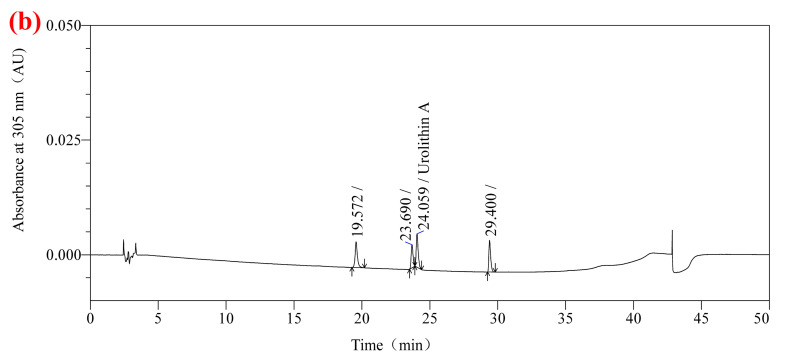
Chromatograms of urolithin A and other impurity peaks (**a**) UHPLC, (**b**) HPLC.

Gradient elution was systematically adjusted to balance analyte retention and separation efficiency. The selected gradient (detailed in Table 3) provided a baseline resolution for urolithin A and its impurities. It is worth noting that, as shown in Figure 1a, the separation factor between the main peak and adjacent impurity peaks exceeds 2.0, reflecting a high degree of specificity. The use of ACQUITY UPLC CSH Fluoro Phenyl (2.1 mm × 50 mm, 1.7 μm) chromatographic column enhanced analyte interactions, contributing to sharper peaks. This chromatographic column helps to effectively separate compounds with similar polarity. Compared to conventional C18 columns, the Fluoro Phenyl column demonstrated higher efficiency for phenolic compounds. As shown in Figure 1b, compared with the method of using HPLC to detect urolithin A, UHPLC can greatly shorten the detection time and achieve better separation than HPLC. Therefore, UHPLC is chosen as the appropriate detection method [17,18].

#### 2.1.2. Diluent Selection

**Figure 2:**
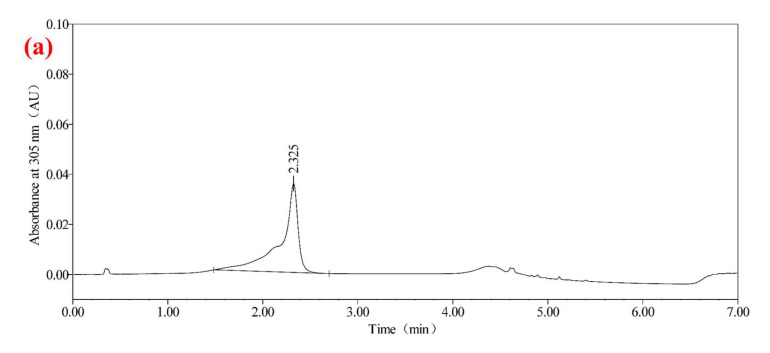

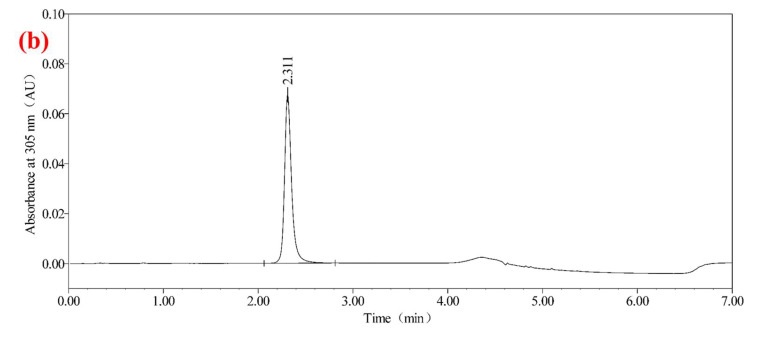
Pure methanol and 40% methanol in water were used for diluent chromatograms: (**a**) chromatogram of pure methanol as diluent; (**b**) 40% methanol aqueous solution as diluent chromatogram.

Diluent choice is essential to maintain analyte stability and improve peak shapes. As shown in Figure 2, testing pure methanol, 40% methanol aqueous solution, and pure water showed that 40% methanol aqueous solution produced optimal chromatographic responses. Peaks were sharp and symmetrical, with no evidence of fronting or tailing. This solvent composition balances solubility and matrix compatibility, crucial for maintaining sample integrity during injection. These findings underscore the importance of aligning diluent properties with the target analyte’s polarity.

### 2.2. Optimization of Sample Extraction Conditions

The efficient extraction of urolithin A is fundamental to achieving accurate quantification. Extraction experiments evaluated solvents (pure methanol, pure water, and methanol–water mixtures) and extraction durations (30, 45, and 60 min). Pure methanol demonstrated the highest recovery rates, achieving over 95% recovery within 30 min. This efficiency is attributed to methanol’s ability to disrupt matrix–analyte interactions while preserving the chemical integrity of urolithin A.

The recovery rates for water and methanol–water mixtures were significantly lower, likely due to their inability to fully dissolve hydrophobic matrix components. Prolonged extraction times (45–60 min) did not enhance recovery, suggesting equilibrium saturation within 30 min. Ultrasonic extraction was chosen for its ability to minimize thermal degradation, a common concern for phenolic compounds.

### 2.3. Specificity

**Figure 3:**
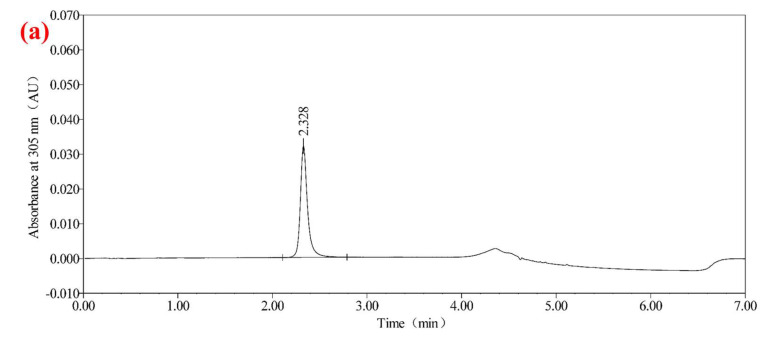

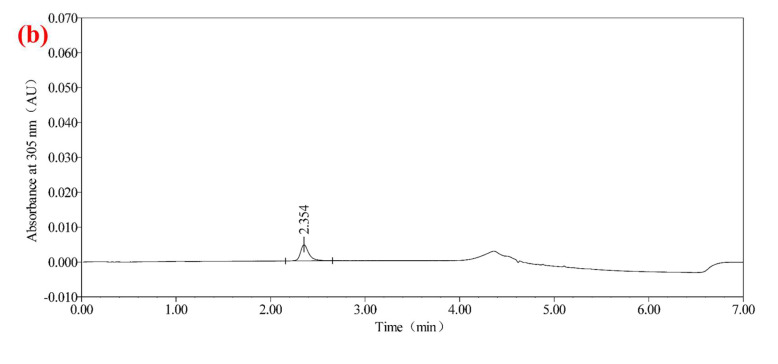

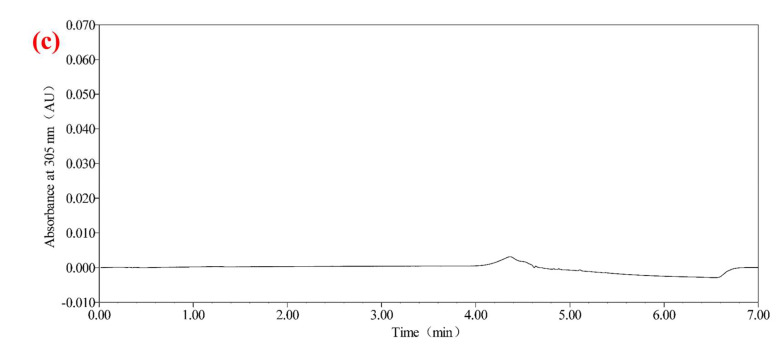
Chromatograms of urolithin A test solution, urolithin A standard working solution (1 μg/mL), and diluent: (**a**) chromatogram of the test solution of urolithin A; (**b**) chromatogram of urolithin A standard working solution; (**c**) diluted liquid chromatogram.

Specificity testing is vital for distinguishing the target analyte from potential interferences. As shown in Figure 3, the analysis of blank, standard, and sample solutions confirmed that the method specifically detected urolithin A without interference from diluent. As shown in Figure 1a, urolithin A peaks were well separated, with resolution factors exceeding the threshold of 2.0. This highlights the robustness of the method in complex matrices, a critical requirement for health product analysis. The resolution between urolithin A and impurity peaks was ≥2.0, with no interference observed in the diluent, meeting the specificity validation requirements of ICH Q2 (R1).

### 2.4. Calibration Curve, Linearity, Detection Limit, and Quantification Limit

#### 2.4.1. Calibration Curve and Linearity

According to the selected chromatographic conditions, the prepared urolithin A standard series working solution is analyzed by ultra-high-performance liquid chromatography, and the chromatographic peak area is recorded. It is necessary to perform linear regression, with the mass concentration of urolithin A standard series working solution as the x-axis (x) and the chromatographic peak area as the y-axis (y), and calculate the linear equation and correlation coefficient. The linear equation for the calculation result is y = 30653x − 2267.4, with a correlation coefficient of 0.9998. The results show that urolithin A exhibits a good linear relationship in the range of 0.100–10.000 μg/mL. Its linearity and range meet the requirements of correlation coefficient ≥0.999 and the range covering, 80~120% of the target concentration in ICH Q2 (R1).

This high degree of linearity is essential for reliable quantification, particularly in quality control settings where trace-level impurities must be accurately detected. Comparatively, the method outperformed traditional HPLC approaches, which often exhibit reduced sensitivity at lower concentrations.

#### 2.4.2. Detection and Quantification Limits

The detection limit refers to the minimum amount of the analyte that can be detected in the sample, while the quantification limit refers to the minimum amount of the analyte that can be quantitatively determined in the sample. This study used the signal-to-noise ratio method to determine the detection limit and quantification limit, with the corresponding concentration at a signal-to-noise ratio (S/N) of 3:1 set as the detection limit and the corresponding concentration at a signal-to-noise ratio (S/N) of 10:1 set as the quantification limit. By continuously diluting the standard stock solution of urolithin A with diluent and injecting it for analysis under selected chromatographic conditions, the detection limit (0.051 μg/mL) and quantification limit (0.103 μg/mL) were significantly lowered compared to industry standards, reflecting the advanced detection capabilities of the UHPLC system. The requirement for quantification limit (RSD ≤ 10%) in ICH Q2 (R1), the result meets the method validation requirements. These parameters enable the method to detect trace levels of urolithin A, critical for health products with stringent regulatory thresholds.

### 2.5. Repeatability

It is necessary to accurately weigh the contents of urolithin A capsules, prepare 6 parallel test solutions according to the method in Section 3.3.1, and inject them according to the liquid chromatography conditions in Section 3.3.2. Then, the scholar must record the peak area and calculate the mass concentrations of urolithin A using the external standard method. In our analysis, the results were 5.589 μg/mL, 5.497 μg/mL, 5.496 μg/mL, 5.449 μg/mL, 5.467 μg/mL, and 5.491 μg/mL, respectively. The average mass concentration was 5.498 μg/mL, with an RSD of 0.9%, thereby demonstrating the method’s precision and meeting the acceptance criteria of RSD < 2% for repeatability. This meets the requirement for repeatability in ICH Q2 (R1) (RSD ≤ 2.0%). These findings confirm the method’s suitability for use in routine analysis in quality control laboratories, where consistency is paramount.

### 2.6. Inter-Operator Precision and Recovery Rate Test

Inter-operator precision was evaluated to determine the method’s robustness under varying operator conditions. Two analysts independently prepared and analyzed six replicates each, yielding an overall RSD of 1.3% and meeting the precision requirements in ICH Q2 (R1) (RSD ≤ 2.0%). The results are shown in Table 1. This low variability underscores the method’s robustness and its applicability across different analysts and laboratories.

It is necessary to prepare 12 portions of urolithin A capsule matrix according to Section 3.3.1, of which, 3 portions are diluted to volume and shaken well. The average value is measured as the background amount. In addition, 9 solutions were sequentially added to standard stock solution 2 (50 μg/mL) at concentrations of 0.8 mL, 0.8 mL, 0.8 mL, 1 mL, 1 mL, 1 mL, 1.2 mL, 1.2 mL, and 1.2 mL, respectively, to prepare 3 sample solutions, each equivalent to approximately 80%, 100%, and 120% of the measured concentration. The sample numbers were A-1, A-2, A-3, B-1, B-2, B-3, C-1, C-2, and C-3. Working according to the chromatographic conditions described above, the content was measured and the recovery rate was calculated. The results are shown in Table 2, indicating that the recovery rate of this method is stable. It also meets the accuracy requirements of ICH Q2 (R1) (98~102%).

### 2.7. Sample Measurement Results

It is necessary to select a commercially available brand of urolithin A health product, prepare the test solution according to the above method, and enter it into an ultra-high-performance liquid chromatography under the chromatographic conditions described above. It is necessary to measure the peak area of urolithin A and calculate the content using a standard curve. The results show that the average content of urolithin A in the sample is 27.5%, and the label value is 27.8%, indicating that this method can be used to detect the content of urolithin A in commercially available health products.

### 2.8. Expanded Discussion

The developed UHPLC method offers several advantages over conventional HPLC techniques, including shorter analysis time, higher sensitivity, and enhanced resolution. Its specificity and robustness make it ideal for the quantification of urolithin A in complex health product matrices. Comparisons with the literature methods revealed that this method achieved superior performance metrics, particularly in terms of detection limits and peak resolution.

Potential applications include routine quality control, regulatory compliance testing, and research on urolithin A bioavailability. However, further validation in diverse matrices, including food and biological samples, is recommended to broaden its applicability.

Moreover, the findings align with global efforts to establish quality standards for bioactive compounds, particularly in emerging markets like China. As the market for anti-aging and functional health products grows, adopting validated analytical techniques will be pivotal in supporting regulatory compliance and consumer trust.

Future research should focus on adapting this method for high-throughput applications and integrating it with advanced detection technologies such as mass spectrometry. These enhancements could further improve detection capabilities, enabling the simultaneous analysis of multiple metabolites in complex biological samples.

## 3. Materials and Methods

### 3.1. Materials and Reagents

The standard reference substance of urolithin A (batch number: D25GB172218, purity 98%) was obtained from Shanghai Yuanye Biotechnology Co., Ltd. (Shanghai, China). Methanol and acetonitrile, both chromatographically pure, were sourced from Merck (Darmstadt, Germany). Ultrapure water was used throughout the study and was purified using a Milli-Q Ultra Pure Water System (Millipore Corporation, Burlington, MA, USA). The health product sample, consisting of urolithin A capsules, was purchased from a verified online retail store.

### 3.2. Instruments and Equipment

Ultra-high-performance liquid chromatography (UHPLC) analyses were conducted using a Waters Acquity Premier UHPLC system (Waters Corporation, Milford, MA, USA), equipped with a UV-visible detector for detection at 305 nm. Chromatographic separation was carried out using an ACQUITY UPLC CSH Fluoro Phenyl column (2.1 mm × 50 mm, 1.7 µm, Waters Corporation, USA). The balance used for precise weighing was a Mettler Toledo XPR205 electronic analytical balance (Mettler Toledo, Zurich, Switzerland, accuracy of 0.0001 g). For sample preparation, an ultrasonic cleaning machine (Emerson, San Francisco, CA, USA), operating at 350 W and 35 kHz, was employed. The separation was performed using a 40% methanol aqueous solution as the dilution solvent and gradient mobile phase composition. Data acquisition and processing were performed with Empower 3 Software (Waters Corporation, USA).

### 3.3. Experimental Methods

#### 3.3.1. Sample Preparation for Urolithin A Capsule (Test Solution)

An appropriate amount of urolithin A capsule content (100.0 mg) was accurately weighed and transferred into a 50 mL volumetric flask. Methanol was added to the flask to cover the sample, and the mixture was subjected to ultrasonic extraction for 30 min at a frequency of 35 kHz and power of 350 W. Following sonication, the solution was allowed to cool to room temperature, and the volume was adjusted with methanol. A 5 mL aliquot of this solution was transferred into a 10 mL centrifuge tube, followed by centrifugation at 11,000 rpm for 10 min to remove particulate matter. The supernatant (0.1 mL) was carefully pipetted into a 10 mL volumetric flask, diluted with 40% methanol aqueous solution, and shaken well. The resultant solution was filtered using a 0.22 µm membrane filter, and the filtrate was retained as the test solution. All samples are prepared and used on site.

#### 3.3.2. Preparation of Capsule Matrix Solution

To prepare the matrix solution, 100.0 mg of the capsule content was weighed and placed into a 50 mL volumetric flask. Following the same procedure as used for the test solution, the sample was sonicated in methanol, diluted to volume, and centrifuged. A 0.01 mL aliquot of the supernatant was then transferred into a 10 mL volumetric flask, further diluted with 40% methanol aqueous solution, and filtered. This preparation was used for matrix interference studies.

#### 3.3.3. Standard Stock and Working Solutions

Urolithin A standard stock solution was prepared by accurately weighing 10.204 mg of the urolithin A reference standard and dissolving it in methanol in a 20 mL volumetric flask. The solution was subjected to ultrasonic extraction until completely dissolved, and the final volume was adjusted to 20 mL, resulting in a concentration of 0.5 mg/mL.

From this standard stock, a working solution (50 µg/mL) was prepared by transferring 11 mL of the stock solution into a 10 mL volumetric flask and diluting with methanol. Several concentrations of urolithin A standard working solutions were prepared by transferring 0.02 mL, 0.2 mL, 0.6 mL, 1 mL, 1.4 mL, and 2 mL of the 50 µg/mL working solution into separate 10 mL volumetric flasks, which were subsequently diluted to the level of 40% methanol aqueous solution to achieve final concentrations of 0.1 µg/mL, 1 µg/mL, 3 µg/mL, 5 µg/mL, 7 µg/mL, and 10 µg/mL, respectively.

### 3.4. UHPLC Analysis Conditions

The chromatographic separation was performed on a Waters ACQUITY UPLC CSH Fluoro Phenyl column (2.1 mm × 50 mm, 1.7 µm) at a column temperature of 40 °C. The mobile phase was composed of two solvents: mobile phase A, which was ultrapure water, and mobile phase B, which was methanol. A gradient elution method was employed, as detailed in Table 3. The flow rate was maintained at 0.4 mL/min, and the injection volume was set at 5 µL. The detector was set to a wavelength of 305 nm for the quantitative determination of urolithin A. All samples were filtered using a 0.22 µm membrane filter before injection to prevent any clogging of the UHPLC column.

**Table 3:** Gradient elution procedure for Urolithin A determination.

Time (min)	Mobile Phase A (%)	Mobile Phase B (%)	Curve
0	60	40	6
3	55	45	6
3.5	1	99	6
5.5	1	99	6
5.6	60	40	6
7	60	40	1

### 3.5. Method Validation

The method was validated for linearity, precision, accuracy, limit of detection (LOD), and limit of quantification (LOQ). A calibration curve was established using the six different concentrations of urolithin A standard working solutions, and the correlation coefficient was determined for the linear range from 0.1 µg/mL to 10 µg/mL. The precision of the method was assessed by repeated injections of the same concentration (n = 6), and the relative standard deviation (RSD) was calculated. Recovery experiments were performed by spiking the capsule matrix solution with known quantities of urolithin A at three different concentrations (low, medium, and high). The average recovery rate and RSD were determined from these experiments.

### 3.6. Data Analysis

The data obtained from the UHPLC analysis were processed using Empower Software (Waters Corporation, USA) for spectral analysis. The concentration of urolithin A in the test samples was determined by comparing the peak areas of the sample chromatograms to the calibration curve. Statistical analysis was performed using Microsoft Office Excel 2010, and RSD values were calculated to assess the method’s reproducibility.

## 4. Conclusions

This study presents a highly effective ultra-high-performance liquid chromatography (UHPLC) method for quantifying urolithin A in health products, addressing the current lack of standardized methodologies for this bioactive compound. By optimizing chromatographic conditions such as the mobile phase, gradient elution, and diluent selection, we developed a method that not only offers superior resolution and specificity but also ensures high sensitivity for the detection of urolithin A, even at trace concentrations. The method demonstrated a strong linear relationship between concentration and peak area, with a correlation coefficient (r^2^) of 0.9998 across a broad concentration range (0.100–10.000 µg/mL). Detection and quantification limits of 0.051 µg/mL and 0.103 µg/mL, respectively, provide the method with an enhanced sensitivity compared to traditional techniques, such as high-performance liquid chromatography (HPLC).

Validation tests confirmed that the method exhibits excellent repeatability, with an RSD of 0.9% for intra-operator precision and 1.3% for inter-operator precision. Recovery tests demonstrated consistent results with average recoveries of around 100.5%, and the method’s specificity was confirmed by the clear separation of urolithin A from its impurities, with resolution factors exceeding the required thresholds. Furthermore, the method was successfully applied to quantify urolithin A content in a commercially available health product, with results closely matching the labeled value, confirming the applicability of the method for routine quality control in the health product industry.

The findings underscore the potential of this UHPLC method for ensuring the safety, efficacy, and regulatory compliance of health products containing urolithin A. This method offers a reliable tool for the quality control of urolithin A in complex matrices and provides an important reference for regulatory bodies, manufacturers, and researchers. Future applications may include broader validation in diverse matrices such as food and biological samples, as well as integration with advanced techniques like mass spectrometry to further enhance its analytical capabilities. Through this work, we contribute to global efforts to establish rigorous quality standards for bioactive compounds in health products, ensuring consumer safety and supporting the growing demand for functional health foods.

## Figures and Tables

**Table 1:** Precision test results (n = 12).

Test Personnel	n	Mass Concentration of Urolithin A (μg/mL)
Tester 1	1	5.589
	2	5.497
	3	5.496
	4	5.449
	5	5.467
	6	5.491
Tester 2	7	5.518
	8	5.378
	9	5.398
	10	5.361
	11	5.387
	12	5.357
average value		5.449
RSD (%)		1.3

**Table 2:** Recovery test results (n = 9).

Background Quantity (μg)	Number	Added (μg)	Total Amount Measured (μg)	Recycling Quantity (μg)	Recovery Rate (%)	Average Recovery Rate (%)	RSD (%)
4.86	A-1	40.00	44.98	40.12	100.3	100.5	0.9
	A-2		45.34	40.48	101.2		
	A-3		45.43	40.57	101.4		
	B-1	50.00	55.52	50.66	101.3		
	B-2		55.35	50.49	101.0		
	B-3		55.40	50.54	101.1		
	C-1	60.00	64.59	59.73	99.6		
	C-2		64.30	59.44	99.1		
	C-3		64.65	59.79	99.7		

## Data Availability

Data are contained within the article.

## References

[1] Tomás-Barberán F.A., González-Sarrías A., García-Villalba R., Núñez-Sánchez M.A., Selma M.V., García-Conesa M.T., Espín J.C. (2016). Urolithins, the rescue of “old” metabolites to understand a “new” concept: Metabotypes as a nexus among phenolic metabolism, microbiota dysbiosis, and host health status. Mol. Nutr. Food Res..

[2] García-Villalba R., Giménez-Bastida J.A., Cortés-Martín A., Avila-Gálvez M.A., Tomás-Barberán F.A., Selma M.V., Espin J.C., González-Sarrías A. (2022). Urolithins: A Comprehensive Update on their Metabolism, Bioactivity, and Associated Gut Microbiota. Mol. Nutr. Food Res..

[3] Espín J.C., Larrosa M., García-Conesa M.T., Tomás-Barberán F. (2013). Biological significance of urolithins, the gut microbial ellagic Acid-derived metabolites: The evidence so far. Evid. Based Complement. Alternat Med..

[4] González-Sarrías A., Giménez-Bastida J.A., García-Conesa M.T., Gómez-Sánchez M.B., García-Talavera N.V., Gil-Izquierdo A., Sánchez-Alvarez C., Fontana-Compiano L.O., Morga-Egea J.P., Pastor-Quirante F.A. (2010). Occurrence of urolithins, gut microbiota ellagic acid metabolites and proliferation markers expression response in the human prostate gland upon consumption of walnuts and pomegranate juice. Mol. Nutr. Food Res..

[5] Lin I.C., Wu J.Y., Fang C.Y., Wang S.C., Liu Y.W., Ho S.T. (2023). Absorption and Metabolism of Urolithin A and Ellagic Acid in Mice and Their Cytotoxicity in Human Colorectal Cancer Cells. Evid. Based Complement. Alternat Med..

[6] Selma M.V., Espin J.C., Tomás-Barberán F.A. (2009). Interaction between phenolics and gut microbiota: Role in human health. J. Agric. Food Chem..

[7] Ares A.M., Toribio L., García-Villalba R., Villalgordo J.M., Althobaiti Y., Tomás-Barberán F.A., Bernal J. (2023). Separation of Isomeric Forms of Urolithin Glucuronides Using Supercritical Fluid Chromatography. J. Agric. Food Chem..

[8] Andreux P.A., Blanco-Bose W., Ryu D., Burdet F., Ibberson M., Aebischer P., Auwerx J., Singh A., Rinsch C. (2019). The mitophagy activator urolithin A is safe and induces a molecular signature of improved mitochondrial and cellular health in humans. Nat. Metab..

[9] D’Amico D., Andreux P.A., Valdés P., Singh A., Rinsch C., Auwerx J. (2021). Impact of the Natural Compound Urolithin A on Health, Disease, and Aging. Trends Mol. Med..

[10] Girotra M., Chiang Y.H., Charmoy M., Ginefra P., Hope H.C., Bataclan C., Yu Y.R., Schyrr F., Franco F., Geiger H. (2023). Induction of mitochondrial recycling reverts age-associated decline of the hematopoietic and immune systems. Nat. Aging.

[11] Hou Y.J., Chu X.X., Park J.H., Zhu Q., Hussain M., Li Z.Q., Madsen H.B., Yang B.M., Wei Y., Wang Y. (2024). Urolithin A improves Alzheimer’s disease cognition and restores mitophagy and lysosomal functions. Alzheimers Dement..

[12] Kujawwska M., Jodynis-Liebert J. (2020). Potential of the ellagic acid-derived gut microbiota metabolite—Urolithin A in gastrointestinal protection. World J. Gastroentero..

[13] Gandhi G.R., Antony P.J., Ceasar S.A., Vasconcelos AB S., Montalvao M.M., de Franca M.N.F., Resende A.D., Sharanya C.S., Liu Y., Hariharan G. (2024). Health functions and related molecular mechanisms of ellagitannin-derived urolithins. Crit. Rev. Food Sci..

[14] Hua Z.Y., Wu Q., Yang Y., Liu S., Jennifer T.G., Zhao D.Y., Fang Y.W. (2024). Essential roles of ellagic acid-to-urolithin converting bacteria in human health and health food industry: An updated review. Trends Food Sci. Technol..

[15] García-Villalba R., Tomás-Barberán F.A., Iglesias-Aguirre C.E., Giménez-Bastida J.A., González-Sarrías A., Selma M.V., Espin J.C. (2023). Ellagitannins, urolithins, and neuroprotection: Human evidence and the possible link to the gut microbiota. Mol. Aspects Med..

[16] Lim RR X., Huang Q.N., Ambrosi A., Bonanni A. (2024). Portable Smartphone-Assisted Graphene Quantum Dots Sensing Platform for the Detection of Gut Microbial Metabolites. ACS Appl. Nano Mater..

[17] de Souza L.P., Alseekh S., Scossa F., Fernie A.R. (2021). Ultra-high-performance liquid chromatography high-resolution mass spectrometry variants for metabolomics research. Nat. Methods.

[18] González-Barrio R., Truchado P., Ito H., Espín J.C., Tomás-Barberán F.A. (2011). UV and MS Identification of Urolithins and Nasutins, the Bioavailable Metabolites of Ellagitannins and Ellagic Acid in Different Mammals. J. Agric. Food Chem..

